# Characteristics of the Properties of Absodan Plus Sorbent and Its Ability to Remove Phosphates and Chromates from Aqueous Solutions

**DOI:** 10.3390/ma15103540

**Published:** 2022-05-15

**Authors:** Eleonora Sočo, Andżelika Domoń, Dorota Papciak, Magdalena M. Michel, Bogumił Cieniek, Dariusz Pająk

**Affiliations:** 1 Department of Inorganic and Analytical Chemistry, Faculty of Chemistry, Rzeszow University of Technology, 35-959 Rzeszow, Poland; eleonora@prz.edu.pl; 2 Department of Water Purification and Protection, Faculty of Civil, Environmental Engineering and Architecture, Rzeszow University of Technology, 35-959 Rzeszow, Poland; dpapciak@prz.edu.pl; 3 Institute of Environmental Engineering, Warsaw University of Life Sciences-SGGW, 02-787 Warsaw, Poland; magdalena_michel@sggw.edu.pl; 4 Institute of Materials Engineering, College of Natural Sciences, University of Rzeszow, Pigonia 1, 35-310 Rzeszow, Poland; bcieniek@ur.edu.pl; 5 Department of Casting and Welding, Faculty of Mechanical Engineering and Aeronautics, Rzeszow University of Technology, 35-959 Rzeszow, Poland; pajak@prz.edu.pl

**Keywords:** chromates and phosphates removal, diatomite, isotherms, kinetics

## Abstract

The aim of the research was to characterize the parameters of the diatomite sorbent Absodan Plus as well as to assess its suitability for the adsorption of chromates and phosphates from acidic aqueous solutions simulating the conditions occurring in some types of industrial wastewater. The scope of the research includes XRD, SEM, BET, and PZC analyses, and 3D observation of commercial diatomite granules and batch tests to determine the constants of kinetics and the equilibrium of chromates and phosphates adsorption. Absodan Plus is a diatomite commercial material containing an amorphous phase (33%) and is also the crystalline phase of quartz, hematite, and grossite. The material is macro- and mesoporous and its specific surface area is about 30 m^2^/g. Its PZC is around pH = 5.5–6.0 and in an acidic environment is able to adsorb the anions. The saturation of the adsorbent surface with molecules of the adsorbed substance occurs after 2 h for chromates and 2.5 h for phosphates. The maximum adsorption capacity of Absodan Plus in terms of phosphorus and chromium amounts to 9.46 mg P/g and 39.1 mg Cr/g, respectively. As shown by XRD analysis, Absodan Plus contains an admixture of hematite, which can support the removal of chromium and phosphorus.

## 1. Introduction

Chromium naturally occurs in the environment (air, water, rocks) and contamination of groundwater and soil with chromates can be of natural origin [1], though anthropogenic pollution is the main problem due to the toxicity and genotoxicity of the chromates [2]. The main emitter of chromium water pollution is the energy sector followed by waste and wastewater management, in EU > 80% and about 10% of total emission respectively [3]. In the energy sector, both thermal power plants and refineries generate chromate releases. As has been shown, the leaching of ashes deposited in the lignite power plant is a hexavalent chromium source and results in high contamination of groundwater (even up to 0.120 ppm) [1]. Industrial wastewater has very diverse characteristics. Electroplating wastewater is very rich in hexavalent chrome (400–2000 ppm) and is strongly acidic (pH 2) [4,5]. The tanning industry also generates chromium-rich wastewater. The production is multistage, and the averaged wastewater has a neutral pH and contains up to several dozen ppm, however, at the pickling and chrome tanning step the acidic wastewater can contain even 2000–3800 ppm of chromium [6,7]. Tannery wastewater can also be slightly alkaline (pH 8) and contain smaller amounts of chromium (several ppm) [8]. Another example of a chromate-contaminated environment is an area of Hanford Site in Washington Stated (USA) [9]. In the production of plutonium in nuclear reactors, a significant amount of chromate has been used as a corrosion inhibitor in reactor cooling water. The deposited chromate sediments and wastes were leached into groundwater and therefore pose an important threat to the environment. Chromite deposits can be the natural source of chromates, as well as anthropogenic, when mining and processing are applied [3]. Especially the poorly managed waste from chromite ore processing is a dangerous factor for the environment [10].

Orthophosphates are a common nontoxic component of the environment. They are desirable for their positive effect on plant cultivation, but their transmission to the aquatic environment has an adverse eutrophication effect [11]. Important point sources of phosphorus pollution are discharges of industrial, municipal, and animal farm wastewater meanwhile, nonpoint pollution is generated by surface runoff from agriculture and urban areas [12,13]. Phosphorus removal from municipal wastewater is efficient at large treatment plants when smaller objects grapple with the lack of effective technology [14]. Even in developed areas of the world, there are still places with no sewage system, from which only partially treated wastewater with nutrients is discharged into the ground [15]. Phosphate rock mining also affects the aquatic environment through runoff of phosphorus-rich drainage waters [16] and phosphate industry processing of raw materials [17]. Industrial wastewater generated in the wet process of phosphoric acid production is characterized by phosphates concentration up to 100 ppm and low pH 2–3 [18,19].

Considering physicochemical techniques, precipitation is used to remove phosphates and chromates from water and wastewater [20] however, it must be noted that during precipitation chromates are additionally reduced to trivalent chromium hydroxide. The wastewater from phosphoric acid production can be treated by precipitation phosphates with ferric and calcium cations even in acidic conditions (pH 3) [18]. Phosphates precipitation connected with ferrous to ferric cation oxidation was also useful to treat wastewater from electroplating in a wide range of pH [21]. Adjustment of pH of neutral pH tannery wastewater to slightly alkaline conditions (pH 8.6–10.3) using NaOH, Ca(OH)_2_, or MgO allows for a very high precipitation efficiency, close to 100% [22]. The same effect was obtained by adjusting acidic tannery effluent to neutral pH using NaOH [23]. Chromates from tannery effluent also can be very effectively coprecipitated with mixed Fe(II) and Fe(III) cations at slightly acidic to neutral conditions (pH 5–7) [8]. However, precipitation technologies can only be beneficial when the resulting sediments are intermediates for further use, even more so when it requires the use of a large dose of reagents in the case of wastewater with extreme pH and high salinity.

Electrocoagulation is a useful method for phosphates removal from acid manufacturing wastewater [19] and chromates removal from tannery wastewater [7] and synthetic wastewater [24] using aluminum and iron electrodes respectively. The ion exchange technique on strongly basic anion resins is one of many chromate removal methods, although due to the sensitivity of the ion exchangers it is used in drinking water treatment [25]. Metal oxides, especially iron oxide, are characterized by the ability to adsorb chromates and phosphates. Iron and manganese oxides, being residues from groundwater treatment, has been evaluated as good adsorbent for chromates removal [26]. The iron precipitates of biogenic origin are also positively assessed for the removal of this pollutant [5]. Iron oxides are often used as coatings on mineral carriers and in this form, they adsorb phosphates [27,28]. The common feature is that the adsorption of chromates and phosphates is intensified under acidic conditions due to electrostatic attraction, ion exchange, and complexation with protonated surfaces [12]. Mineral materials such as indigenous volcanic rock [29], aluminosilicate rock containing feldspar, montmorillonite, and illite [30], diatomite, and zeolite [31], dolomite and opoka [32], zeolitic tuff [33] are also used to remove chromates and phosphates.

Diatomite is a siliceous sedimentary rock with a predominant opal content and a smaller amount of quartz, calcite, clay minerals, iron compounds, or glauconite [34]. Its biogenic nature comes from the shells of diatoms that build diatomite and give the rock a porous structure. The lithological variability between deposits and even within one deposit results in differentiated porosity, density, specific surface area, and adsorption capacity of diatomite [35,36,37]. As a result, diatomite sorbents may have different properties depending on their origin. Meso- and macroporosity is a characteristic feature of this material, due to which it is widely used as a filtration aid in many industrial and environmental applications and the porosity can be additionally increased by heat processing [38,39] or acid treatment [40]. Diatomite is used in various industrial applications as a filler, carrier, holder, sorbent, abrasive, and ingredient [35].

The known use of diatomite is the production of diatomaceous sorbents for the removal of oil spills and other petroleum contaminants [41,42]. Diatomite can also be used as a filling in biosorption filters as it stimulates the growth of the nitrifying biofilm [42,43]. Diatomite has the ability to adsorption of cations by ion exchange and electrostatic attraction with dissociated silanol groups [44]. In this way, the raw diatomite can remove Pb, Cu, Cd, Ni, and Ag cations from water solutions [39,45,46]. In an acidic environment, the surface of diatomite undergoes protonation and as a result, it is able to adsorb oxyanions like chromate, phosphate, and arsenite [32,47,48,49,50]. This proves the possible use of diatomite in the treatment of wastewater containing chromates and phosphates. Other studies show that opal-A is of little importance for the adsorption of arsenates, but there is a correlation between the effectiveness of their removal and the content of iron oxide being the natural impurities of the rock [51]. This is an important premise, indicating that the parent rock admixtures have a significant impact on their adsorption properties, therefore particular commercial diatomite sorbents may have different effectiveness. The unique properties of diatomite enable its widespread and multi-sector use. The USA (35%) and China (19%) dominate the world diatomite market, but the EU produces around 13% of the global product [52]. Diatomite sorbents for general purposes are popular in the trade. They are dedicated to the removal of pollutants from the environment as well as to protecting the environment against contamination from entering it. Commercial universal diatomite sorbents are designed to remove liquid substances such as oils, fuels, paints, inks, acids, bases, water coolants, and other solvents from industrial floors [53,54,55]. Research on the use of diatomite for heavy metal removal is important because it is an abundant, low-cost eco-environmental functional material [38,56]. There are many studies on the sorption on diatomites from various quarries [35,36,39,40,42,48,49,56], but these are not commercial products. The popular diatomite sorbent Absodan Plus (AP) is intended for a wide range of applications [55], however, research was carried out in the field of removing petroleum derivatives oil [57]. Also, it is important to determine the sorption capacity of commercial diatomites for other non-standard applications (not specified by the manufacturer), as these materials are potentially available.

The aim of the research was to characterize the material parameters of the commercial diatomite sorbent Absodan Plus as well as to assess its suitability for the adsorption of chromates and phosphates from acidic aqueous solutions simulating the conditions occurring in some types of industrial wastewater. The scope of the research includes: X-ray diffraction (XRD), scanning electron microscopy (SEM) analysis, Brunauer-Emmett-Teller (BET) surface area analysis, point of zero charge (PZC) analysis, and 3D observation of commercial Absodan Plus granules as well as batch tests to determine constants of kinetics and equilibrium of chromates and phosphates adsorption.

## 2. Materials and Methods

### 2.1. Adsorbent

#### 2.1.1. Characteristics of Absodan Plus

The diatomite sorbent Absodan Plus (Damolin, Katowice, Poland) was used for the tests, which, according to the safety data sheet, is calcined diatomaceous earth. Its main application is the removal of liquid substances such as fats, greases, oils, fuels, and water from the ground. It is also designed to absorb bases, acids, solvents, and impurities from water solutions. An exception is a hydrofluoric acid due to the large amounts of SiO_2_. Absodan Plus is nonflammable, harmless, chemically inert, and odorless. It has many advantages, as it does not release the absorbed liquid, has a high absorption capacity of up to 130% of its weight, and allows multiple uses [55]. The chemical composition of the tested diatomite is presented in Table 1.

#### 2.1.2. Analysis of Absodan Plus Properties

A detailed study was conducted to evaluate Absodan Plus’s structure, chemical composition, and properties. The samples of diatomite were subjected to the following set of analyses:

- X-ray diffraction analysis (XRD): The mineral composition of diatomite was assessed using X-ray diffraction with CuK_α1_ radiation, with scan step 0.015 degrees, scan rate 2 s/step, and scan range from 17 to 70° 2θ at 40 kV and 40 mA (Bruker D8 Advance, Bruker AXS, Germany). The average crystallite sizes (D) of samples were calculated from the XRD data by applying the Debye–Scherrer Equation: D = 0.89λ/BcosΘ, where λ is the wavelength of the X-ray in nanometres, B is the peak width at half-height (FWHM), and θ is the angle between the incident and diffracted beams in angular degrees.
- Crystallinity and amorphous % of diatomite from its scan were computed with Bruker Eva software, as follows:
  % Amorphous = ((Global area − Reduced area)/Global area) × 100 % Crystallinity = 100 − % Amorphous.
- SEM microscopic analysis: The morphological and textural observation of the surface was made by scanning electron microscope (SEM) (TESCAN VEGA 3, Fuveau France). SEM was used also with a back-scattered electron detector (BSE) (INCA x-act, Oxford Instruments) to broaden the scope of the element content analysis.
- Brunauer-Emmett-Teller (BET) surface area analysis: Specific surface area and total pore volume were determined using low-temperature nitrogen adsorption-desorption isotherms using an ASAP 2020 porosimeter (Micromeritics, Norcross, GA, USA). Before measurements, the samples were degassed at 200 °C. The specific surface area and total pore volume were calculated by the Brunauer-Emmett-Teller (BET) method and the Barrett-Joyner-Halenda (BJH) method, respectively.
- Stereoscopic image of the diatomite was analyzed by X2000 series microscopes (Opta-Tech, Warsaw, Poland) are designed for observation of small, 3D objects in transmitted and reflected light, and the 2D surface of diatomite measurements of topography, and layer thickness were carried out by confocal 3D microscope (NanoFocus, Oberhausen, Germany).
- Fourier transform infrared spectroscopy (FTIR) analysis: FTIR spectra of Absodan Plus were obtained with an Alpha spectrometer (Bruker, Billerica, MA, USA). The tests were carried out with the transmission method, using the technique of pressing samples with potassium bromide. Compressed adsorbent samples were mixed with KBr at a constant adsorbent weight to KBr weight ratio of 0.25% and pelleted under pressure. The FTIR spectra were employed in a spectral range of 4000 to 500 cm^−1^ [58].
- Determination of point of zero charge (PZC) of diatomite: The PZC was determined using three methods:
  1. Suspension method: In a series of 250-mL Erlenmeyer flasks, 0.5 g of diatomite was added to 50.0 mL of 0.1 M NaNO_3_ solution. The pH was adjusted with 0.1 M HNO_3_ and 0.1 M NaOH as needed, to obtain the appropriate pH values of 2, 3, 4, 5, 6, 7, 8, 9, 10, and 11. The samples were shaken for 12 h using a shaker: vibration amplitude 8.5; rate 180 (ELPIN PLUS, type 358 A, Lubawa, Poland). After settling, the pH values of the supernatant in each flask were again measured. After settling, the pH values of the supernatant in each flask were again measured [59,60].
  2. Potentiometric method: Three flasks containing 0.05 g of diatomite, 3 mL of 0.1 M NaNO_3_ (to establish an ionic strength constant), and 1 mL of 0.01 M NaOH were added to 6 mL of distilled water. Then all samples were titrated with 0.01 M HCl. Based on the obtained data, a plot of the dependence of the potential change [mV] on the volume of titrant consumed [mL] was made. The research was carried out for a blank and a test sample. The diatomite potential was measured using a combination glass electrode (Elmetron, type OSH 10-00, Zabrze, Poland) [59,60].
  3. Hahn’a Method: The method consists in determining the highest increase of the potential (∆*E*_max_) and two adjacent potential values that lie on both sides of it (∆*E*_1_, ∆*E*_2_). Based on these values, the correction (*x_b_*) is calculated, which increases the accuracy of the PZC determination (the correction enables the precise determination of the volume of the titrant at the place of its occurrence [61]. The calculations were done according to Equation (1):
    (1) $$x_{b}=\frac{\Delta V\cdot \Delta E_{2}}{2\cdot \Delta E_{1}}$$
    where:
    *x_b_*—correction,
    ∆*V*—a volume of titrant,
    ∆*E*1—a potential gain that occurs before ∆*E_max_*.

### 2.2. Adsorbate

The sorption of phosphates and chromates on diatomite was studied by batch experiments. The concentration of phosphates and chromates after the sorption process was determined using a UV-VIS spectrophotometer (JASCO, model V-670) at the wavelength λ = 720 nm and two absorption maxima of 350 nm and 373 nm.

**Figure 1:**
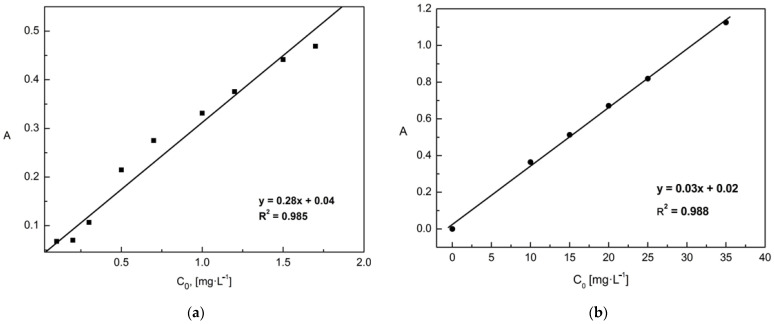
Determined appropriate absorption coefficient (ε) necessary for the determination concentration of: (**a**) phosphates ions and (**b**) chromates ions.

- Determination of the concentration of phosphates ions by the molybdenum blue method: The standard curve was prepared based on a series of phosphates ions solutions with concentrations ranging from 0 to 1.7 mg/L. The absorbance of the prepared solutions was measured against the reagent blank (sample without the addition of phosphates ions). Immediately before the measurement, 1.5 mL of ammonium molybdate and 2 drops of tin (II) chloride (reducing agent in the reaction of the formation of navy blue molybdenum blue) were added to each of the samples. Because the molybdenum method cannot determine strongly acidic or basic aqueous solutions, all samples were neutralized by adding six drops of 6 M NaOH each. The standard curve equation was determined by linear regression using the least-squares method. The determined proper absorption coefficient (ε), corresponding to the slope in the straight line equation, was 0.28 L/mg·cm (Figure 1a).
- Determination of the concentration of chromates ions by the chromate method (UV/VIS spectrophotometry): Absorbance measurements were performed for given standard solutions with known concentrations in the range of 0 to 35 mg/L. The absorbance of the prepared solutions was measured against the blank reagent (a sample without the addition of chromates ions. The determined proper absorption coefficient (ε) was 0.03 L/mg·cm (Figure 1b).

### 2.3. Batch Studies of the Adsorption Process

#### 2.3.1. Effect of Shaking Time on Adsorption

0.5 g of diatomite and 50 mL of a solution of phosphates or chromates ions at a concentration of 50 mg/L were added to 6 flasks with a capacity of 100 mL. The pH of the solutions was adjusted to 2 with 6 M HNO_3_ to create conditions typical of acidic industrial wastewater [34,35,48,49]. The prepared samples were shaken for 15–400 min, then the suspension obtained was filtered on a filter paper. The content of phosphates or chromates was determined in the supernatant.

#### 2.3.2. Effect of Adsorbent Concentration on Adsorption

0.5 g of diatomite and 50 mL of previously prepared solutions of phosphates or chromates (pH 2) with concentrations of 10, 50, 200, 350, 400, 1000, 2000, and 3000 mg·L^−1^ were added to 5 flasks with a capacity of 100 mL. Then they were placed on a shaker, and adsorption was carried out for 1.5 h. After this time, the resulting mixture was filtered on a filter paper, and the content of phosphates or chromates was determined in the filtrate.

### 2.4. Models of Equilibrium and Kinetics of Adsorption

To fully understand the adsorption nature of phosphates or chromates ions in commercial diatomite sorbent, graphs of Freundlich, Langmuir, Halsey, Jovanovich, and Redlich-Peterson isotherms were prepared [Table 2] [62]. On their basis, it was determined which isotherm equation describes the studied phenomenon most accurately. The coefficient of determination (R^2^) and the chi-square statistic reduced by the number of degrees of freedom (χ^2^/DoF) were used to define the fit of the models to the results of the experiment. The calculations were made in Origin 7.5. The kinetic evaluation of the realized adsorption process was also performed with the use of pseudo-first and pseudo-second-order kinetic models [Table 3].

To determine the rate-limiting stage of the adsorption process, the intraparticle diffusion model developed by Weber and Morris [63] was used. This model is presented in the Table 3.

## 3. Results and Discussion

### 3.1. Adsorbent Characteristics

**Figure 2:**
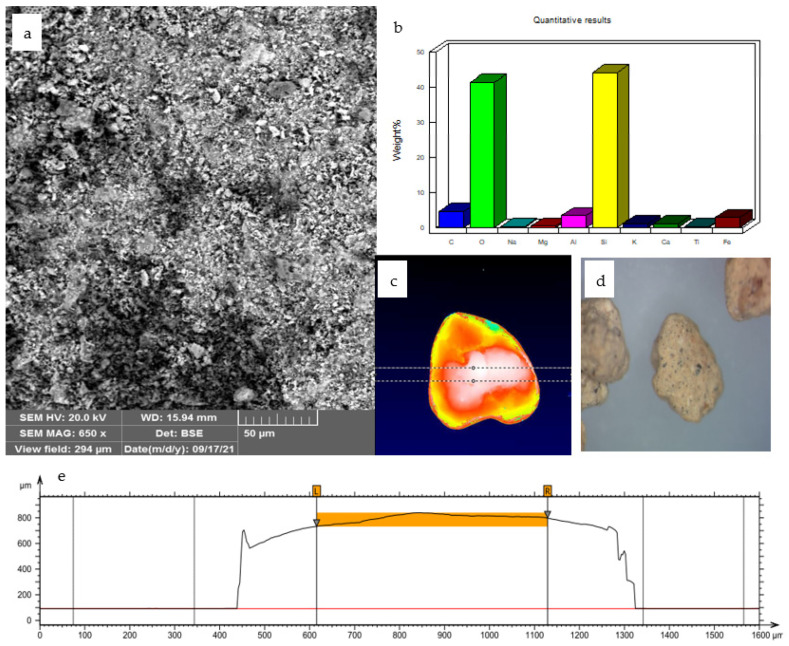
Absodan Plus: (**a**) SEM surface micrograph (650×), (**b**) content of identified elements diatomite, (**c**) 2D image (**d**) stereoscopic image, (**e**) topography layer (extracted profile: maximum height 747 μm, mean height 710 μm).

**Figure 3:**
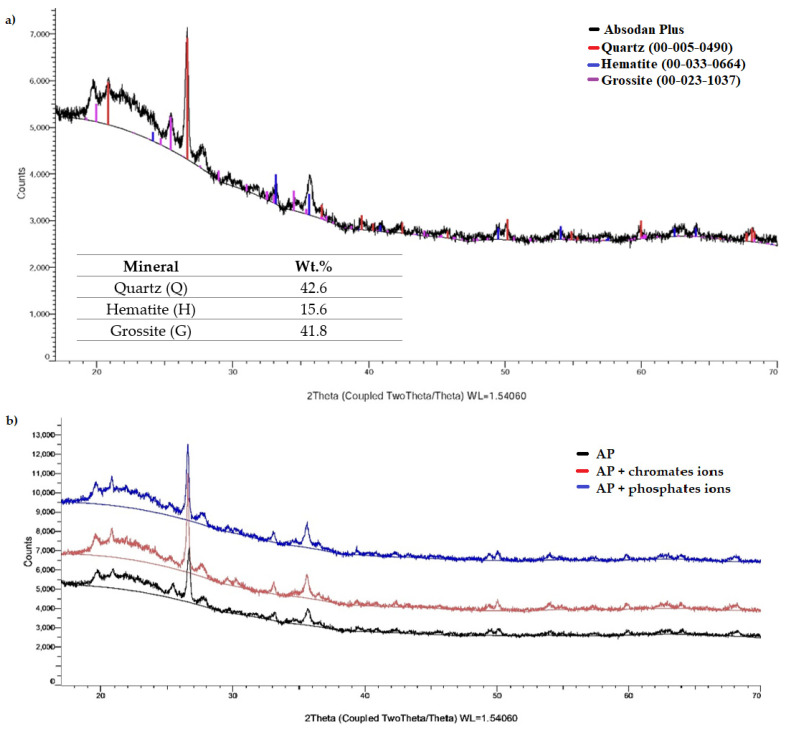
XRD diffractograms of: (**a**) Absodan Plus, (**b**) Absodan Plus with chromates and phosphates ions.

**Figure 4:**
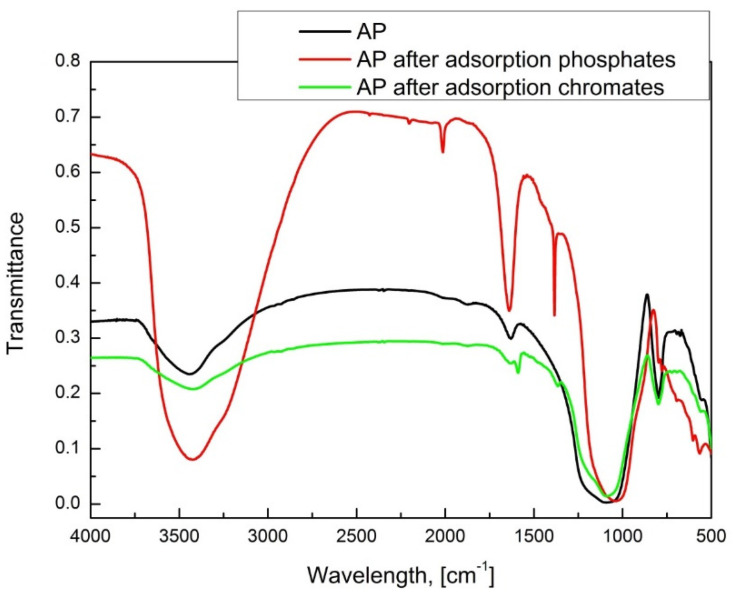
FTIR analysis of Absodan Plus samples.

SEM microscopic observations allowed to characterize the morphology of the diatomite (Figure 2). The surface composed of mesopores and macropores indicates high porosity and low density of this material, which was also observed in previous studies [57]. The results of the BET analysis show that the specific surface area of the Absodan Plus was 30.6 m^2^/g, while the total pore volume was 0.46 cm^3^/g. The stereoscopic and two-dimensional 2D image of diatomaceous earth together with the layer thickness is shown in Figure 2. XRD analysis of Absodan Plus showed that quartz, hematite, and grossite are the main minerals of the crystalline phase. They were found approximately in the 2.73:1:2.68 ratio, respectively (Figure 3). As a result of the adsorption of chromates and phosphates ions, the structure of the diatomite slightly changes. XRD analysis performed confirms the change in the size of the crystallites and grains of the adsorbent tested (Figure 3). A strong diffraction peak (1 0 1) at 26.60° 2θ comes from quartz (Figure 4). Based on this peak, the crystallite size (D) was calculated for three samples (Table 4). The change in crystallite size is not significant and remains in the same order of magnitude. Crystallinity and amorphous ratio are shown in Table 4. Diatomite adsorbent is characterized by a twice higher proportion of the crystalline phase as compared to the amorphous phase. The presence of phosphate and chromate adsorbates does not significantly affect the proportion of these phases.

The FTIR spectrum was performed to identify functional groups on the surface of the adsorbent used. As a result, it was possible to observe the structural changes caused by the ongoing adsorption process. This is especially important from the point of view of regenerating this type of adsorbent, and thus the possibility of its multiple uses in subsequent processes. The spectrum analysis is shown in Table 5.

In all the spectra of the tested samples of Absodan Plus, there are characteristic bands in the following areas: 3440 cm^−1^, 1630 cm^−1^, 1050 cm^−1,^ and 800 cm^−1^ (Figure 4). The band around 3443 cm^−1^ can come from both the hydroxyl groups that build the water molecule and those that have been bonded to the AP surface by chemical bonds. The band located at the value of 1643 cm^−1^ indicates the presence of C=C bonds and carboxyl-carbonate structures. The most intense of the bands located at 1076 cm^−1^ comes from vibrations of C-O bonds that occur in ether, carboxyl, and phenol groups. The band at the value of 796 cm^−1^ is the result of the presence of SiO_4_^4−^ and AlO_4_^5−^ systems in the tetrahedral samples, forming the three-dimensional structure of diatomites [72].

In the spectra of the samples after the adsorption process, an additional band appears at the wavenumber value of 1386 cm^−1^. It was suspected that it could be derived from an adsorbed orthophosphate (V) ion or a complex formed by it. To verify the thesis, the experimental spectrum was correlated with the FTIR spectrum of sodium dihydrogen phosphate, the source of phosphate ions in the conducted research. It turned out that there is a characteristic band in the range of 1300–1400 cm^−1^. This fact confirms that the 1386 cm^−1^ band is derived from molecules of the adsorbate attached to the AP surface (Figure 4). After analyzing the spectroscopic spectra, it was found that the process of adsorption of phosphate ions and, to a lesser extent, chromate ions take place.

**Figure 5:**
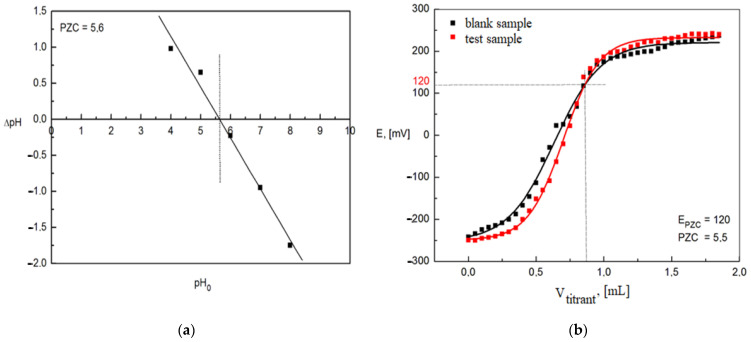
PZC of the adsorbent: (**a**) suspension method; (**b**) potentiometric method.

The value of the zero point of the adsorbent load determined by the suspension method was 5.6 (Figure 5a). PZC is the point of intersection of the plot of the dependence ∆pH = f (pH 0) with the OX axis, therefore it is equivalent to the zero point of this function. In the case of the potentiometric method, the PZC was 5.5 (Figure 5b). The value was read from the graph of the dependence of the potential change [mV] on the volume of used titrant [mL]-PZC is located at the intersection of the lines on the graphs. PZC determined by Hahn’s method was 5.9 [Table 6], its average value fluctuates around pH = 5.5–6.0, i.e., in the area classifying the reaction of the environment as slightly acidic. The pH of the aqueous diatomite solution was 5.2, which is below the zero point of the electric charge. This means that the Absodan Plus surface is positively charged, and it has a greater ability to attach anions than cations. Speciation of phosphorus and chromium anions is strongly pH-dependent. The experiment was carried out in an acidic environment (pH 2), and in the range of the concentrations used, the anions were not fully dissociated. Phosphorus was present as H_3_PO_4_ and H_2_PO_4_^−^ with a slight quantitative predominance of the undissociated form [73]. Under these conditions, the dominant form of chromate was partially dissociated HCrO_4_^−^ (~80%) and in a dozen or so percent Cr_2_O_7_^2−^ [74], as well as the possible content of undissociated acid H_2_CrO_4_ [75]. This allows the conclusion that the conditions of the experiment are favorable because a positively charged surface would interact with the anions as a result of electrostatic attraction [75].

### 3.2. The Adsorption Equilibrium

**Figure 6:**
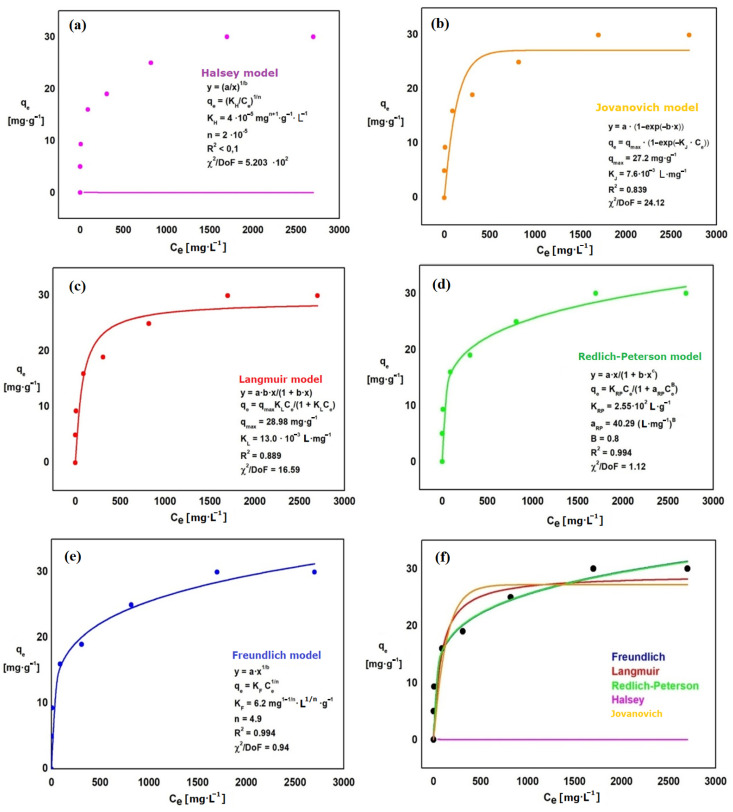
Isotherms: Halsey (**a**), Jovanovich (**b**), Langmuir (**c**), Redlich-Peterson (**d**), Freundlich (**e**), comparison of isothermal models for adsorbed phosphates ions (**f**).

**Figure 7:**
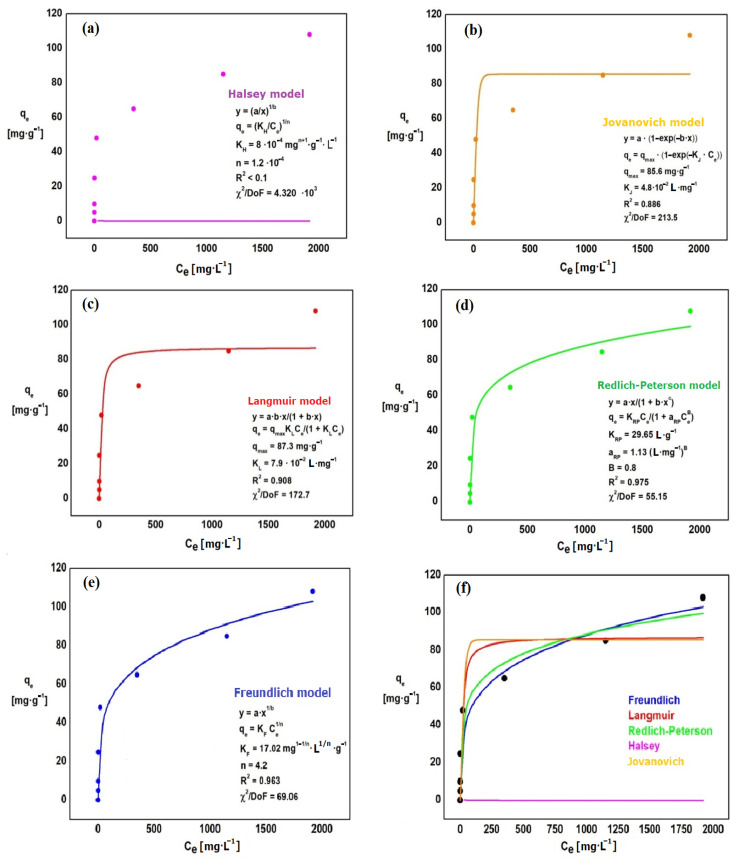
Isotherms: Halsey (**a**), Jovanovich (**b**), Langmuir (**c**), Redlich-Peterson (**d**), Freundlich (**e**), comparison of isothermal models for adsorbed chromates ions (**f**).

The adsorption isotherms of phosphates ions (Figure 6) and chromates ions (Figure 7) were prepared to select the model that best describes the adsorption process on Absoban Plus. Each of the isotherms is represented by the relation *q_e_* = *f*(*C_e_*). The following isotherms were made: Halsey, Jovanovich, Langmuir, Redlich-Paterson, and Freundlich. The comparisons of isothermal models for adsorbed phosphates and chromates ions are presented in Figure 6 and Figure 7. The parameters obtained for all isothermal models for adsorbed chromates and phosphates ions are presented in Table 7.

A good fit to the experimental points is represented by those isotherms that determine the relationship *q_e_* = *f*(*C_e_*). The parameters determined from each of these equations can help assess the adsorption efficiency, the affinity of the adsorbent for the adsorbate, and whether the sorption system used is favorable or not.

The Redlich-Peterson isotherm was the best fit for the experimental points. This is confirmed by the calculated coefficient of determination, which for this model has the highest value: R^2^ = 0.975 (chromates ions) and 0.994 (phosphates ions). The error in fitting the theoretical curve to the experimental data also reaches the smallest value (χ^2^ = 6.0 and 8.5, respectively) (Table 7). The parameters of this equation are, in the case of chromium ions, respectively: K_RP_ = 29.7 L·g^−1^, a_RP_ = 1.13 L·mg^−1^ and B = 0.8. For phosphates ions, the parameters assume the following values: K_RP_ = 255 L·g^−1^, a_RP_ = 40.3 L·mg^−1^ and B = 0.8 (Table 7). The worst fit shows the Halsey isotherm, for which the value of R^2^ is <0.1 and the value of χ^2^/DoF is 520 (phosphates ions) and 4320 (chromates ions). The Halsey model is used for multilayer adsorption on the heterogeneous surface [76], which does not occur with the adsorption performed.

The $\frac{1}{n}$ parameter in the Freundlich isotherm characterizes the surface of the adsorbent. The values should be between 0 and 1. The closer the value to $\frac{1}{n}$ is to 0, the more ideal the adsorbent surface is. On the other hand, values of $\frac{1}{n}$ close to 1 define the surface as heterogeneous. In the experiment, the value of n was determined at 4.2 (chromates ions) and 4.9 (phosphates ions), and $\frac{1}{n}$ at 0.24 (chromates ions) and 0.20 (phosphates ions). To predict whether sorption under certain conditions is favorable, the partition coefficient R_L_ can be calculated for each measuring point. R_L_ is characteristic of systems described by the Langmuir isotherm. When the R_L_ is between 0–1, then adsorption on selected components is favorable. If R_L_ > 1, it means that the system has unfavorable sorption characteristics for the phenomenon studied. The relationship is calculated based on the following equations:

(2) $$R_{L}=\frac{1}{1+a_{L}C_{0}}$$

(3) $$a_{L}=\frac{K_{L}}{q_{\max}}$$

where:

R_L_—partition coefficient;
a_L_—the quotient of the equilibrium constant for the isotherm and the maximum area coverage for the model.

**Figure 8:**
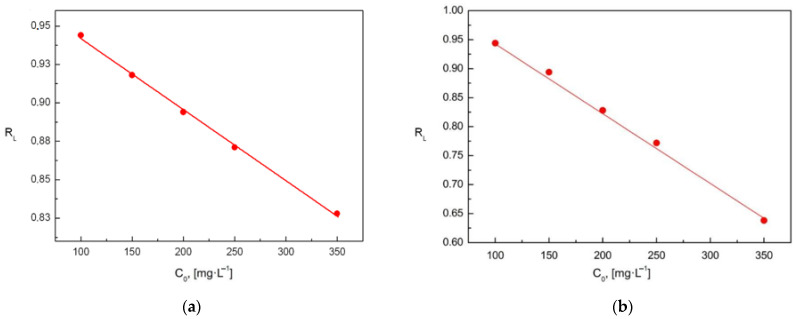
Dependence of the equilibrium parameter R_L_ on the initial concentration of: (**a**) chromates ions and (**b**) phosphates ions in aqueous solutions for the Langmuir isotherm.

For the analyzed adsorption system, a graph of R_L_ = *f*(*C*_0_) was prepared (Figure 8). It shows that the calculated parameter is in the range from 0 to 1. This means that the surface phenomenon related to the removal of the examined ions is favorable.

The Langmuir maximal adsorption capacity of Absodan Plus converted to phosphorus is 9.46 mg P/g. It is rather a high capacity compared to other capacities obtained for natural diatomites and ranges from 0.46–3.51 mg P/g (Table 7). The Absodan Plus adsorption capacity converted to chrome is 39.1 mg Cr/g. It is a very good result compared to capacities of calcined diatomite and raw Carpathian diatomite equal to 0.2 mg Cr/g and 0.12 mg Cr/g respectively [48,49]. As shown in Table 8, the adsorption capacity of Absodan Plus is satisfactory compared to unmodified diatomites. Various modifications lead to an increase in the efficiency of removing of phosphates and chromates by the transformed surface of the diatomite. A much higher removal capacity can be achieved by lanthanum oxide modified diatomite [77] or MCM-41 composite with refined diatomite containing a higher concentration of diatom frustules [50]. However, they are more advanced materials that require special reagents to produce. It is also a mineral that effectively adsorbs phosphates by surface complexation and precipitation [78,79,80]. As shown in Table 8 diatomite coated by hydrous iron oxide and metallic iron/iron oxides are characterized by a significant increase in the adsorption capacity of phosphates and chromates [27,28]. Other studies have shown that hematite can adsorb chromates, especially in an acidic environment [81]. As shown by the XRD analysis, Absodan Plus contains an admixture of hematite, which can support the removal of chromium and phosphorus.

### 3.3. The Adsorption Kinetics

**Figure 9:**
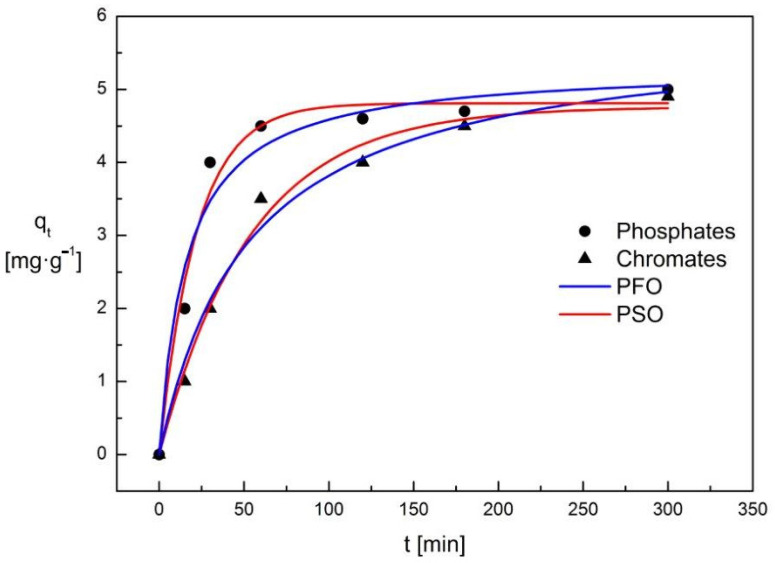
The pseudo-first-order and pseudo-second-order kinetic curves for the adsorption process for chromates and phosphates ions on an Absodan Plus.

When the adsorbent surface is saturated with molecules of the adsorbed substance, the adsorption capacity deteriorates, and the process should be stopped. Changes in phosphate ions concentration in solution and on the surface of the adsorbent during the adsorption process are shown in Figure 9. During the adsorption process, there is an exponential increase in the concentration of phosphate ions immobilized on the surface of the Absodan Plus and a decrease in their concentration in the solution. The concentration of the tested ions on the adsorbent surface initially increases rapidly, and after about 2 h, it practically does not change. Initially, the decrease in the concentration of phosphate ions is significant, then it is slowed down to a constant value after about 2.5 h. The situation is similar in the case of chromate ions (Figure 9). The obtained results indicate that chromate ions had easier access to the active centers of diatomite, which was confirmed by the size of the analyzed ions (phosphate ions are higher than chromate ions).

To investigate the mechanism and determine the rate of the adsorption process, pseudo-first-order (PFO) and pseudo-second-order (PSO) kinetic models were developed (Table 9). Both the adsorbent and the adsorbate molecules could participate in the adsorption of chromates and phosphates ions on the diatomite. The analysis of pseudo kinetic curves showed that they were not linear (the kinetic equation is not fulfilled). The pseudo-second-order kinetic curves showed linear dependencies that go through the origin of the coordinate system (Figure 9). The pseudo-second-order kinetic equation has been met, as evidenced by a very good fit to the experimental data (R^2^ values equal to 1). Three factors influence adsorption: the adsorbent, water, and the ions dissolved in it.

**Figure 10:**
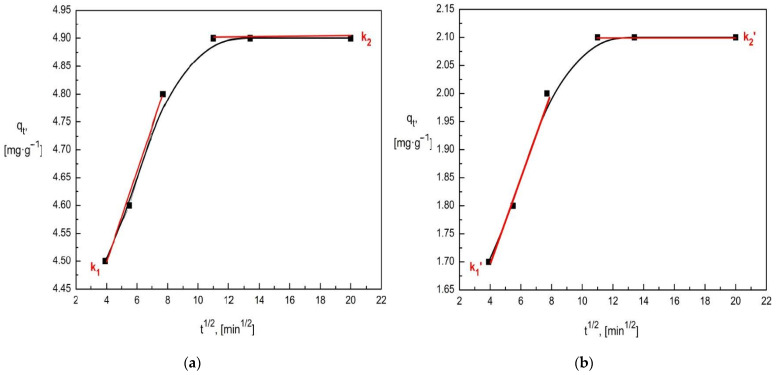
Intraparticle diffusion model in the diatomite adsorption process: (**a**) chromates ions, (**b**) phosphates ions.

To determine the rate-limiting stage of the adsorption process, the intraparticle diffusion model developed by Weber and Morris [63] was used (Figure 10). The adsorption process can be divided into two stages: surface diffusion and intraparticle diffusion (mass transport takes place in each stage). The surface diffusion that occurs through the boundary layer is characterized by a high rate. Then the substance migrates deep into the structure of the diatomite, slowly filling the available pores until it fills their entire volume. The Weber model allows us to determine which of these stages affects the overall rate of adsorption [63].

The non-linear course of the entire adsorption process confirms that it is multistage. For the linear fragments in the diagrams (Figure 10), additional graphs were prepared and simple equations were determined. The slope values in the equations are equal to the diffusion process rate constant (Table 10). The first segment in the graphs is assigned to boundary layer diffusion, which is the main rate-limiting step for the entire process. The higher the value of the intercept in the equation of these lines, the greater the effect of boundary layer diffusion on the overall rate of the adsorption process. The second segment shows intraparticle diffusion. The slope values in the simple equations fitted to this stage correspond to the intraparticle diffusion constant (k_1_, k_2_), which in both cases is close and almost equal to zero. This is because this adsorption step is almost a constant function and does not significantly affect the rate of the entire process.

## 4. Conclusions

The Absodan Plus is a diatomite commercial material containing an amorphous phase (33%) and also the crystalline phase of quartz, hematite, and grossite. The material is macro and mesoporous and its specific surface area is about 30 m^2^/g. Its PZC is around pH = 5.5–6.0 and in an acidic environment is able to adsorb the anions. The saturation of the adsorbent surface with the adsorbed anions occurs after 2 h for chromates ions and 2.5 h for phosphates ions. The results obtained indicate that chromate ions had easier access to the diatomite’s activity centers. Among the analyzed models of isotherms, the Redlich-Peterson isotherm is the best fit for the experimental points (R_2_ = 0.975 and 0.994 for chromates and phosphates ions, respectively) showed. The rate-limiting stage of the adsorption of chromates and phosphates ions on diatomite is diffusion in the boundary layer. Intraparticle diffusion slightly affects the kinetics of the process. The maximum adsorption capacity of Absodan Plus in terms of phosphorus and chromium amounts to 9.46 mg P/g and 39.1 mg Cr/g, respectively. The adsorption capacity of Absodan Plus is satisfactory compared to unmodified diatomites. Various modifications lead to an increase in the phosphate and chromate removal efficiency by the reshaped diatomaceous earth surface. As shown by XRD analysis, Absodan Plus contains an admixture of hematite, which can support the removal of chromium and phosphorus.

## Figures and Tables

**Table 1:** Physicochemical properties of the tested diatomite—Absodan Plus (safety data sheet) [based on ref. [55]].

Parameter	Value	Parameter	Value
SiO_2_, % (*w*/*w*)	75	MgO, % (*w*/*w*)	2
Al_2_O_3_, % (*w*/*w*)	7	K_2_O + Na_2_O, % (*w*/*w*)	2
Fe_2_O_3_, % (*w*/*w*)	9	Residue on ignition (1025 °C), % (*w*/*w*)	2
TiO_2_, % (*w*/*w*)	1	Weight density, g/mL	2.3
MnO_2_, % (*w*/*w*)	1	pH (10% suspended solids)	5.5
CaO, % (*w*/*w*)	1	Bulk density, g/L	509

**Table 2:** Lists of adsorption isotherm models.

Isotherms	Equation	Abbreviations	References
Freundlich	$q_{e}=K_{F}\cdot {\left(C_{e}\right)}^{\frac{1}{n}}$	q_e_—the equilibrium concentration of the adsorbate on the adsorbent surface [mg·g^−1^], K_F_—Freundlich adsorption equilibrium constant [mg^1−1/n^·L^1/n^·g^−1^], C_e_—the concentration of the adsorbate in the solution at equilibrium [mg·L^−1^], n—adsorption intensity, q_max_—maximum adsorption capacity [mg·g^−1^], K_L_—Langmuir adsorption equilibrium constant [L·mg^−1^], a_R_—Redlich-Peterson constant [L·mg^−1^]^βR^, K_R_—Redlich-Peterson adsorption equilibrium constant [L·g^−1^], βR—parameter dependent on the concentration of the adsorbate, K_J_—Jovanovich adsorption equilibrium constant [L·g^−1^], K_H_—Halsey adsorption equilibrium constant [mg^n+1^·g^−1^·L^−1^].	[64,65]
Langmuir	$q_{e}=q_{\max}\cdot \frac{K_{L}\cdot C_{e}}{1+K_{L}\cdot C_{e}}$		[65,66,67]
Jovanovich	$q_{e}=q_{\max}\cdot \left(1-e^{\left(K_{J}\cdot C_{e}\right)}\right)$		[68]
Halsey	$q_{e}={\left(K_{h}\cdot C_{e}\right)}^{\frac{1}{n}}$		[69,70]
Redlich-Peterson	$q_{e}=\frac{K_{R}\cdot C_{e}\;}{1+a_{R}\cdot C_{e}^{\beta R}}$		[64,65,66,67]

**Table 3:** Lists of kinetic models [71].

Kinetic Model	Pseudo-First-Order (PFO)	Pseudo-Second-Order (PSO)	Intra-Particle Diffusion
Equation	dq/dt = k_1_(q_e_ − q_t_) q_t_ = q_e_(1 − e^−k_1_t^)	dq/dt = k_2_(q_e_ − q_t_)^2^ q_t_ = k_2_q_e_^2^t/(1 + k_2_q_e_t)	q_t_ = k_i_t^1/2^ + b_i_
where: q_t_—adsorption capacity (in time t) [mg·g^−1^], k_i_—the intra-particle diffusion rate constant for adsorption [mg·g^−1^·min^1/2^], b_i_—the boundary layer thickness [mg·g^−1^].			

**Table 4:** Crystallinity and the amorphous ratio of Absodan Plus take into account the size of crystallites.

Sample	Crystallite Size, Å	Crystallinity, %	Amorphous, %
**AP**	342	66.6	33.4
**AP + chromates ions**	552	66.0	34.0
**AP + phosphates ions**	408	68.6	31.4

**Table 5:** Analysis of the FTIR absorption spectrum of Absodan Plus samples-characteristic bands.

Wavelength, [cm^−1^]	Band Description	The Band Comes from:
3443	Very wide, high intensity	the stretching vibrations of the –OH group.
1643	Narrow, low intensity	the stretching vibrations of the groups: C=C, CO-CH_2_-CO
1386	Very narrow, low intensity	the adsorbed PO_3_^4−^ ion
1076	Broad, high intensity	the stretching vibrations of the C-O groups
796	Narrow, low intensity	the stretching vibrations of asymmetric C-H groups; band located in the dactyloscopy area

**Table 6:** Values from the titration table based on which the PZC was determined by Hahn’s method.

Parameter	∆E1	∆Emax	∆E2	pH_0.8_ *
**Value**	63.4 mV	52.8 mV	43.4 mV	5.9

* index 0.8 is the volume of added titrant at the PZC point, [mL].

**Table 7:** Isotherm parameters for the adsorbed phosphates and chromates ions.

Phosphates Ions					Chromates Ions				
**Halsey isotherm**									
K_H_ [mg^n+1^·g^−1^·L^−1^]	n	R^2^		X^2^/DoF	K_H_ [mg^n+1^·g^−1^·L^−1^]	n	R^2^		X^2^/DoF
4·10^−5^	2·10^−5^	<0.1		520	8·10^−4^	1.2·10^−4^	<0.1		4320
**Jovanovich isotherm**									
K_J_ [L·mg^−1^]	q_max_ [mg·g^−1^]	R^2^		X^2^/DoF	K_J_ [L·mg^−1^]	q_max_ [mg·g^−1^]	R^2^		X^2^/DoF
0.008	27.2	0.839		24.1	0.048	85.6	0.886		213
**Langmuir isotherm**									
K_L_ [L·mg^−1^]	q_max_ [mg·g^−1^]	R^2^		X^2^/DoF	K_L_ [L·mg^−1^]	q_max_ [mg·g^−1^]	R^2^		X^2^/DoF
0.013	28.9	0.889		16.6	0.079	87.3	0.908		173
**Freundlich isotherm**									
K_F_ [mg^1−1/n^·L^1/n^·g^−1^]	n	R^2^		X^2^/DoF	K_F_ [mg^1−1/n^·L^1/n^·g^−1^]	n	R^2^		X^2^/DoF
6.2	4.9	0.994		0.94	17.0	4.2	0.963		69.1
**Redlich-Peterson isotherm**									
K_RP_ [L·g^−1^]	a_RP_ [L·mg^−1^]	B	R^2^	X^2^/DoF	K_RP_ [L·g^−1^]	a_RP_ [L·mg^−1^]	B	R^2^	X^2^/DoF
255	40.3	0.8	0.994	1.12	29.7	1.13	0.8	0.975	55.2

**Table 8:** The comparison of the adsorption capacity of Absodan Plus with capacities of various diatomaceous sorbents.

	Material	Maximal Adsorption Capacity, mg/g	Ref.
**P**	diatomite	0.46 mg P/g	[37]
	diatomite	0.60 mg P/g	[28]
	diatomite	3.51 mg P/g	[27]
	hydrous Fe oxide modified diatomite	5–25 mg P/g	[28]
	diatomite coated by Fe^0^ and Fe oxides	37.0 mg P/g	[27]
	La oxide modified diatomite	58.7 mg P/g	[77]
	Absodan Plus	9.46 mg P/g	present study
**Cr**	calcined diatomite	0.20 mg Cr/g	[47]
	diatomite	0.12 mg Cr/g	[75]
	Fe oxide modified diatomite	6.10 mg Cr/g	[47]
	diatomite-MCM-41 composite	70.9 mg Cr/g	[50]
	Absodan Plus	39.1 mg Cr/g	present study

**Table 9:** Kinetic PSO and PFO model constants and correlation coefficients for the sorption systems.

Parameter	Pseudo-First-Order (PFO)		Pseudo-Second-Order (PSO)	
	Phosphates Ions	Chromates Ions	Phosphates Ions	Chromates Ions
χ^2^/DoF	0.26	0.18	0.06	0.02
R^2^	0.953	0.979	0.989	0.993
k_1_ [min^−1^]	1.3·10^−2^	2.8·10^−3^	4.8·10^−2^	2.1·10^−2^
q_e_ [mg·g^−1^]	5.5	5.7	4.2	4.9

**Table 10:** Values of the diffusion rate constants of the phosphates and chromates adsorption process.

The Type of the Process	Line Equation	The Value of the Diffusion Rate Constant [mg/g·min^1/2^]
Boundary layer diffusion for chromates ions	$q_{t}=8\cdot 10^{-2}\cdot t^{\frac{1}{2}}+4.2$	$k_{1}=8\cdot 10^{-2}$
Intraparticle diffusion for chromates ions	$q_{t}=2.7\cdot 10^{-15}\cdot t^{\frac{1}{2}}+4.9$	$k_{2}=2.7\cdot 10^{-15}$
Boundary layer diffusion for phosphates ions	$q_{t}=8\cdot 10^{-2}\cdot t^{\frac{1}{2}}+1.4$	$k_{1}^{’}=8\cdot 10^{-2}$
Intraparticle diffusion for phosphate ions	$q_{t}=2.5\cdot 10^{-15}\cdot t^{\frac{1}{2}}+2.1$	$k_{2}^{’}=2.5\cdot 10^{-15}$

## Data Availability

Not applicable.
